# Lipid Metabolic Reprogramming in Thyroid Cancer: Systemic Biomarkers, Tumor Dependencies and Therapeutic Resistance

**DOI:** 10.3390/metabo16100736

**Published:** 2026-09-30

**Authors:** Li Zhou, Yiqing Mi, Linlin Mao, Jingjing Shi, Yu Zhang, Gang Pan, You Peng

**Affiliations:** 1 Department of Surgical Oncology, Affiliated Hangzhou First People’s Hospital, School of Medicine, Westlake University, No. 261 Huansha Road, Shangcheng District, Hangzhou 310006, China; zhouli@hospital.westlake.edu.cn (L.Z.); maolinlin@hospital.westlake.edu.cn (L.M.); jingjingshi123@163.com (J.S.); zhangyu89@zju.edu.cn (Y.Z.); pangang@hospital.westlake.edu.cn (G.P.); 2 The Fourth Clinical Medical College, Zhejiang Chinese Medical University, No. 548 Binwen Road, Binjiang District, Hangzhou 310053, China; 202411122511052@zcmu.edu.cn

**Keywords:** thyroid cancer, lipid metabolism, lipidomics, fatty acid metabolism, cholesterol, SCD1, ferroptosis, immunometabolism, therapeutic resistance

## Abstract

**Highlights:**

**What are the main findings?**

- Thyroid cancer lipid reprogramming involves three interconnected but distinct layers: circulating lipid markers, tumor-intrinsic lipid flux, and immune-spatial metabolic niches.
- Fatty-acid uptake and oxidation, SCD1-dependent desaturation, cholesterol remodeling, and ferroptosis defense are linked to tumor progression and treatment resistance, although most evidence remains preclinical and histotype-specific.

**What are the implications of the main findings?**

- Circulating lipid levels should not be interpreted as direct measures of intratumoral lipid dependency or as evidence that nonspecific lipid-lowering therapy will control thyroid cancer.
- Translation should prioritize biomarker-guided combination strategies, paired tumor–blood metabolic profiling, and prospective evaluation of toxicity and metabolic compensation.

**Abstract:**

Lipid metabolic reprogramming is increasingly implicated in thyroid cancer progression and treatment resistance alongside circulating lipid associations are often conflated with tumor lipid dependency. A clinically useful synthesis must distinguish systemic lipid markers from intratumoral lipid flux before integrating them into mechanistic or therapeutic models. This critical narrative review integrates evidence across three connected layers: circulating lipid markers; tumor-intrinsic uptake, oxidation, synthesis, desaturation, cholesterol and oxysterol pathways; and immune-spatial niches involving macrophage lipid handling, APOE-associated polarization and ferroptosis-related lipid peroxidation. In papillary thyroid carcinoma, multi-omics and mechanistic studies implicate LPL-FATP2-CPT1A, PC-AKT/mTOR-SREBP1c-FASN and METTL16/YTHDC2/SCD1 programs in membrane remodeling, invasion-related behavior and recurrence risk stratification. In aggressive and anaplastic thyroid cancer, cholesterol synthesis signaling, fatty acid oxidation adaptation and SREBF1/SCD1-linked ferroptosis defense may contribute to resistance to radioiodine, radiotherapy, chemotherapy and BRAF/MEK inhibition. The current evidence is strongest for mechanistic and biomarker hypotheses; thyroid-cancer-specific prospective trials of lipid-directed treatment are lacking. Lipid metabolism therefore provides a framework for molecular stratification and treatment sensitization, but it does not currently justify nonspecific lipid lowering or routine lipid-targeted therapy. Translation will require paired blood and tumor lipidomics, spatial profiling, functional perturbation and biomarker-defined prospective studies.

## 1. Introduction

Thyroid cancer comprises papillary thyroid carcinoma (PTC), follicular thyroid carcinoma (FTC), medullary thyroid carcinoma (MTC), poorly differentiated thyroid carcinoma (PDTC) and anaplastic thyroid carcinoma (ATC). PTC accounts for most cases and dominates the current metabolic evidence base. PTC and FTC are differentiated thyroid cancers (DTCs) that usually retain more thyroid lineage function and may respond to radioactive iodine, whereas PDTC and ATC show progressive dedifferentiation, aggressive behavior and treatment resistance. MTC originates from parafollicular C cells and is biologically distinct. These differences preclude assuming that a lipid dependency identified in PTC also operates in undifferentiated or C-cell-derived disease. Integrated proteogenomic and metabolomic studies further show substantial heterogeneity even within clinically defined PTC recurrence risk groups [1].

The state of the art has progressed through three overlapping phases. Early metabolomic and lipidomic studies focused on diagnostic or prognostic signatures; multi-omics studies then resolved recurrence-associated lipid modules and tumor-versus-adjacent-tissue differences; and recent perturbation studies have begun to connect fatty acid oxidation, lipogenesis, desaturation, cholesterol synthesis and ferroptosis defense to invasion or treatment adaptation [1,2,3,4,5,6,7]. Despite this progress, most evidence remains observational or preclinical, and no lipid-directed treatment is established specifically for thyroid cancer.

Recent reviews have summarized thyroid cancer metabolomics and lipidomics, lipid metabolism disorders, lipidomic profiling and fatty acid metabolic reprogramming [2,3,4,5]. However, systemic dyslipidemia, tumor-intrinsic lipid flux, immune cell lipid handling and ferroptosis are still frequently treated as partially separate fields of research. This separation can encourage an unsupported inference that a circulating lipid concentration directly measures, or causes, a tumor metabolic dependency.

The present critical narrative review addresses this gap by integrating evidence across three layers: systemic lipid markers; tumor-intrinsic fatty acid, cholesterol, oxysterol and lipid peroxidation programs; and immune-spatial lipid niches. It evaluates evidence by histotype and study design, distinguishes associative biomarkers from functionally supported vulnerabilities, and prioritizes clinically testable sensitization strategies for recurrence, dedifferentiation and resistance to radioiodine, radiation and MAPK pathway therapy.

## 2. Review Approach and Evidence Appraisal

We conducted a critical narrative review with structured evidence mapping rather than a PRISMA systematic review or meta-analysis. PubMed was searched on 25 May 2026, public trial and omics repositories were scanned on 26 May 2026, PubMed was refreshed on 5 June 2026, Wanfang Data and VIP/CQVIP were searched on 7 June 2026, and the clinical trial scan was refreshed on 12 June 2026. The searches covered thyroid cancer combined with fatty acid uptake and oxidation, de novo lipogenesis, desaturation, cholesterol and oxysterol metabolism, lipidomics, immunometabolism, ferroptosis and therapeutic resistance. Full search strings, screening categories, counts and audit files are provided in Supplementary File S1.

We prioritized patient tissue, blood-based and spatial omics studies; mechanistic studies with genetic or pharmacologic perturbation, rescue experiments, xenograft models or patient-derived organoids; and clinical cohorts or causal inference studies relevant to circulating lipid markers. Bioinformatic signatures, network pharmacology and prior reviews were used mainly for context or hypothesis generation. Evidence was not considered clinically mature without thyroid-cancer-specific prospective intervention or biomarker validation data.

To make the evidence categories reproducible, each chain in Table 1 was scored across five domains from 0 to 2 points: thyroid/histotype directness (D), human relevance (H), functional causality (C), model depth (M) and independent replication of the same mechanism (R). A score of 0 denotes absent or indirect support, 1 denotes partial support, and 2 denotes strong support within each domain, yielding a total of 0–10. The detailed anchors for every score are provided in Supplementary File S1. This is an author-designed narrative framework, not formal GRADE, because the evidence base consists mainly of omics, observational biomarker and preclinical mechanistic studies rather than comparative intervention trials.

Total scores of 0–3 were categorized as emerging and 4–6 as moderate. The suffix ‘preclinical’ indicates that functional perturbation contributed to the score but prospective clinical validation is absent. A moderate–high preclinical categorization required a score of 7–10, direct thyroid cancer evidence, functional perturbation, model depth and independent support; a mechanistically deep single study was capped at moderate preclinical categorization. Clinically established categorization was reserved for prospective thyroid-cancer-specific validation and was not reached by any chain. Table 1 also reports the number of independent publications directly supporting each broad evidence chain; contextual reviews and reused datasets were not counted as independent studies.

The supplementary resource scan identified trial signals and reusable omics resources but was not used for formal systematic inclusion. No thyroid-cancer-specific prospective anticancer trial of statins, ketogenic intervention, SCD1 inhibition, FAO inhibition, GPX4 inhibition or ferroptosis-directed lipid targeting was identified. One active first-in-human phase I study is evaluating the SCD1 inhibitor MTI-301 across refractory metastatic or unresectable solid tumors (NCT06911008), but no safety or efficacy results are available and the study is not thyroid-cancer-specific. Embase, Web of Science, Scopus, CNKI and SinoMed were not counted as having been searched because completed reproducible searches were not obtained.

During manuscript preparation, the authors used OpenAI Codex (GPT-5; OpenAI, accessed May–August 2026) to support language polishing. The authors independently verified study eligibility, source data, citation placement, evidence interpretation and final wording, and take full responsibility for the manuscript.

The synthesis is organized by histotype and evidentiary layer. Table 1 reports the score, category and independent study count for each evidence chain. Table 2 ranks candidate interventions by translational priority and specifies the application, potential toxicity, anticipated adaptation and next validation step. Figure 1 maps the systemic, tumor-intrinsic and immune-spatial layers, whereas Figure 2 summarizes sensitization opportunities and barriers.

The figure separates systemic lipid inputs from tumor cell lipid programs and immune-spatial niches. Circulating LDL, HDL, TG and apoB are presented as systemic biomarkers or sources of substrate availability, not as direct measurements of intratumoral lipid flux. APOC1 and APOE are represented within the apolipoprotein program node. The dashed connection from circulating markers denotes an associative or context-dependent relationship, whereas solid arrows denote more direct metabolic or functional evidence. In tumor cells, an LPL-FATP2-CPT1A candidate chain is linked to fatty acid uptake and FAO, while PC-AKT/mTOR-SREBP1c-FASN, METTL16/YTHDC2/SCD1 and YTHDF2/SREBF1/SCD1 represent candidate lipid synthesis, desaturation and RNA-regulated programs. Cholesterol and oxysterol nodes include LDLR, CYP27A1, CYP7B1, 27-HC and RTN3-DHCR7-dependent cholesterol synthesis. Ferroptosis defense is represented by SLC7A11-GSH-GPX4 and therapy-induced lipid peroxidation. Immune and spatial features include CD36-positive macrophages, APOE-associated M2 polarization, SPP1/PI3K-AKT tumor cell interaction and spatial metabolomics/transcriptomics across primary tumors, adjacent tissue and lymph node metastases. These processes converge on proliferation, migration/invasion, metastasis and recurrence risk, dedifferentiation, aggressive behavior and resistance to RAI, radiotherapy, chemotherapy and BRAF/MEK inhibitors.

The figure presents lipid metabolic intervention as a stratification and sensitization hypothesis rather than nonspecific lipid lowering or established therapy. The clinical context column covers PTC progression/recurrence, BRAFV600E adaptation, MEK inhibitor insensitivity, DTC/RAIR and ATC resistance, and immune metabolic niches. The strategy column maps these contexts to blockade of lipid supply and synthesis, suppression of an FAO-driven adaptive state, targeting of an RTN3-DHCR7-HMGCR cholesterol synthesis candidate pathway, induction of lipid peroxidation/ferroptosis, and modulation of CD36/APOE/SPP1-related immune lipid handling. The clinical maturity column distinguishes preclinical, organoid-supported and emerging biomarker hypotheses. The banner notes that no thyroid-cancer-specific prospective lipid-targeting trial was identified while acknowledging the active pan-solid-tumor phase I MTI-301 study. The key barrier column identifies patient selection, on-target safety, metabolic compensation, dose tolerance, tumor selectivity, resistance bypass, trial–evidence gaps, drug interactions, biomarker selection, normal-tissue safety, timing and sequence, subtype specificity, functional causality, spatial validation and normal immune function.

## 3. Histotype Context and Conceptual Framework

Thyroid cancers do not share a uniform lipid phenotype. Differentiated thyroid cancers (DTCs), dominated by PTC and including FTC, generally retain more lineage differentiation and may remain responsive to radioactive iodine, whereas PDTC and ATC progressively lose thyroid-specific functions and rely more heavily on stress-adaptation programs. MTC arises from parafollicular C cells and represents a distinct lineage. The available lipid metabolism literature is therefore histotype-skewed: PTC supports the most detailed multi-omics and mechanistic synthesis, ATC contributes a smaller but treatment-resistance-focused body of research, and evidence in FTC, PDTC and MTC remains sparse. Mechanisms should not be transferred across these groups without direct validation.

We organize the evidence across three connected but non-equivalent layers. The systemic layer includes circulating lipids, lipoproteins and inflammatory–lipid ratios that may reflect host metabolism, tumor burden or reverse causation. The tumor-intrinsic layer includes fatty acid acquisition, synthesis, desaturation, oxidation, cholesterol handling and lipid peroxidation defense. The immune-spatial layer captures lipid handling by macrophages and metabolically distinct tumor niches. Figure 1 uses a dashed link for systemic associations and solid links for more direct metabolic or functional relationships.

This histotype and evidence hierarchy provides the storyline for the sections that follow. We first examine tumor-intrinsic lipid programs, then ask how these programs interact with immune-spatial niches and treatment stress, before returning to the more clinically accessible but less mechanistically direct blood biomarkers and, finally, we provide a prioritized translational roadmap.

## 4. Tumor-Intrinsic Lipid Programs

Tumor-intrinsic evidence is strongest in PTC and ranges from moderate multi-omics associations to moderate–high preclinical support for selected desaturation pathways. The central question is not whether one lipid enzyme is uniformly increased, but how thyroid cancer cells balance exogenous lipid acquisition, endogenous synthesis, mitochondrial oxidation and membrane remodeling as parallel and compensatory routes.

### 4.1. Fatty Acid Acquisition, Oxidation and Membrane Remodeling in PTC

One important feature of lipid metabolic change in PTC is the reorganization of the fatty acid supply chain rather than the isolated upregulation of a single enzyme. Multi-omics analysis comparing PTC with adjacent thyroid tissue demonstrated clear separation of tumor and non-tumor lipid profiles [6]. Tumors showed decreased triglycerides and diacylglycerols, together with increased membrane lipid classes such as phosphatidylcholine, phosphatidylinositol, phosphatidylglycerol, phosphatidylethanolamine, sphingomyelin and ceramides. This pattern suggests that fatty acids can be mobilized from storage lipids and redirected toward membrane remodeling, signaling or mitochondrial utilization.

At the molecular level, this work proposed an LPL-FATP2-CPT1A candidate chain. LPL hydrolyzes triglycerides to release fatty acids, FATP2/SLC27A2 supports fatty acid transport, and CPT1A is a rate-limiting enzyme for mitochondrial beta-oxidation of long-chain fatty acids. LPL, FATP2 and CPT1A were increased in PTC tissue and were associated with clinical progression or survival measures [6]. Functional experiments further showed that overexpression of these molecules enhanced thyroid cancer cell migration. Although migration assays do not by themselves establish an in vivo metastatic cascade, they move the LPL-FATP2-CPT1A candidate chain beyond biomarker association and toward a functionally testable metabolic program.

An earlier Chinese patient tissue study provides independent expression-level support for related fatty acid handling. After array-based discovery in three paired PTC and adjacent tissue samples, RT-PCR validation in 46 paired samples showed higher tumor expression of SLC27A6/FATP6 and FABP3 [8]. This finding broadens the transporter and fatty-acid-binding candidates implicated in PTC, but it remains supportive rather than causal because functional perturbation and outcome validation were not reported.

This fatty acid supply chain model is consistent with recurrence risk multi-omics. In 102 Chinese patients with PTC, high-risk disease showed altered triglycerides, free fatty acids and metabolic modules [1]. Integrated clustering identified a high-recurrence risk metabolic subtype with poor outcome, suggesting that lipid metabolism contributes to risk stratification within PTC rather than merely separating tumors from adjacent tissue.

Additional studies link fatty acid metabolism to BRAF-driven adaptation. In BRAFV600E PTC, ACC2 was downregulated in clinical samples and cell lines, while BRAF inhibition altered de novo lipid synthesis, FAO and metabolic flux [9]. More recent work connected FAO to epigenetic therapy adaptation: BRAFV600E inhibition increased FAO and PGC1alpha, and FAO-derived acetyl-CoA was linked to H3K9ac remodeling and RUNX1-dependent survival programs. FAO inhibition with thioridazine enhanced the antitumor effect of BRAF inhibition in vitro, in vivo and in patient-derived organoids [10]. Other nodes include hypoxia–CPT1A signaling [11], circPCNXL2-ACC1-dependent fatty acid metabolism [12] and combined reliance on LPL-mediated exogenous uptake and FASN-mediated endogenous synthesis [13]. Collectively, these data suggest that PTC can mobilize exogenous uptake, endogenous synthesis and mitochondrial oxidation, and may rebalance these routes under therapeutic pressure.

Related serum and plasma studies have identified free fatty acid, metabolite and lipid panels with diagnostic potential [14,15]. Because these circulating signatures do not measure intratumoral flux, their clinical interpretation is deferred to the dedicated systemic-biomarker section.

### 4.2. De Novo Lipogenesis: A Proposed PC-AKT/mTOR-SREBP1c-FASN Pathway

In addition to the uptake and utilization of exogenous or stored fatty acids, thyroid cancer cells may support aggressive phenotypes through de novo lipogenesis. Pyruvate carboxylase (PC) catalyzes an anaplerotic reaction that converts pyruvate to oxaloacetate, thereby supplying carbon for biosynthesis. A mechanistic study proposed that a PC-AKT/mTOR-SREBP1c-FASN pathway may support thyroid cancer aggressiveness [16].

PC was increased in invasive PTC compared with normal thyroid tissue in public transcriptomic data and was further associated with lymph node metastasis in 38 PTC surgical samples [16]. PC knockdown reduced intracellular triglycerides, lipid droplets and free fatty acids, including oleic acid, stearic acid, palmitoleate and palmitic acid. It also downregulated FASN, ACC1 and ACLY, as well as SREBP1c. In clinical tissues, PC correlated with FASN and SREBP1c mRNA expression. Mechanistically, PC knockdown reduced AKT and mTOR phosphorylation, and PI3K inhibition decreased SREBP1c and lipid accumulation. Functionally, PC knockdown inhibited migration, invasion and xenograft growth, whereas SREBP1c overexpression partially rescued lipid accumulation, invasion and tumor weight.

This proposed pathway links tricarboxylic acid cycle anaplerosis, lipid synthesis and invasive behavior in a single experimentally testable model. It complements, rather than replaces, the LPL-FATP2-CPT1A model: the latter emphasizes mobilization, uptake and oxidation, whereas the PC-centered pathway emphasizes endogenous lipogenesis. Because the detailed PC-AKT/mTOR-SREBP1c-FASN chain is supported primarily by one multilevel study, we classify it as moderate preclinical evidence despite its rescue and xenograft data. Independent replication, larger molecularly annotated cohorts and pharmacologic target-engagement studies are required before clinical translation.

### 4.3. SCD1 and Epitranscriptomic Control of Desaturation

Whereas the PC-centered pathway emphasizes the metabolic activation of fatty acid synthesis, the METTL16/YTHDC2/SCD1 findings suggest that PTC lipid reprogramming can also be controlled at the epitranscriptomic level. SCD1 converts saturated fatty acids into monounsaturated fatty acids and thereby affects membrane composition, triglyceride synthesis, proliferation and migration. Because tumor cells require lipid synthesis and membrane remodeling, SCD1 is a plausible metabolic adaptation node.

A mechanistic study of 58 paired PTC and adjacent tissues integrated TCGA/cBioPortal analysis, RNA sequencing, m6A sequencing, fatty acid and triglyceride assays, cell function experiments and mouse tumor models [17]. METTL16 was decreased in PTC, potentially through DNMT1-mediated promoter hypermethylation. METTL16 overexpression inhibited PTC proliferation, migration, invasion and in vivo metastasis, whereas METTL16 knockdown had the opposite effect. The key finding was that METTL16 increased m6A modification in the 3′ untranslated region of SCD1 mRNA, while YTHDC2 recognized the modification and was reported to promote SCD1 mRNA degradation. Loss of METTL16 therefore stabilized SCD1 and enhanced lipid accumulation.

Metabolic assays supported this mechanism: METTL16 overexpression reduced palmitate, oleic acid, triglycerides and Oil Red O staining, whereas METTL16 knockdown increased lipid accumulation [17]. Pharmacologic inhibition of SCD1 with A939572 suppressed proliferation, colony formation, migration, triglyceride accumulation and tumor growth, and partially reversed the effects of METTL16 knockdown. This positions SCD1 as a potential vulnerability in METTL16-low PTC. Additional evidence links SCD to immune infiltration and PTC cell invasion [18], and early ATC work identified aberrant lipid metabolism and SCD1 as a therapeutic target [19]. In 50 paired PTC samples and two PTC cell lines, KMT5A was overexpressed, while its knockdown reduced SREBP1, SCD, FASN and ACC expression together with MDA, ROS and malignant cell phenotypes [20]. TCGA data from 455 PTCs further associated high KMT5A with extrathyroidal extension, lymph node metastasis and advanced stage. These findings support chromatin-associated regulation of a lipogenic program, but they do not establish the proposed p53-SREBP intermediary mechanism, and lipid flux or in vivo therapeutic effects were not tested.

SCD1 targeting in thyroid cancer remains preclinical. Systemic SCD1 inhibition has raised mechanistic and preclinical concerns because the enzyme supports lipid homeostasis in normal tissues, including skin and ocular surface tissues. The active first-in-human MTI-301 phase I study in refractory solid tumors is designed to define dose and toxicity and excludes patients with clinically relevant corneal disease or grade 2 or greater neuropathy (NCT06911008); these criteria indicate safety domains under surveillance, not established treatment-emergent toxicities. No clinical results are available. Compensation through exogenous fatty acid uptake, CPT1A-mediated oxidation or alternative lipogenic pathways also remains a major concern and should be built into future combination studies.

### 4.4. Cholesterol Uptake, Oxysterol Metabolism and Targeted-Therapy Adaptation

Cholesterol metabolism is another major component of thyroid cancer lipid reprogramming. Cholesterol supports membranes and lipid rafts, and oxysterol derivatives can modulate inflammation, proliferation and migration. However, cholesterol metabolism in thyroid cancer should not be interpreted simply as “higher serum cholesterol means higher cancer risk.” A more plausible model is that aggressive tumors increase cholesterol uptake and intracellular cholesterol handling, thereby altering both circulating lipid profiles and intratumoral oxysterols.

An integrated clinical and mechanistic study included benign thyroid tumors, low/intermediate-risk PTC, high-risk PTC and PDTC/ATC, and measured 27-hydroxycholesterol (27-HC) in frozen tumor samples [21]. High-risk PTC and PDTC/ATC were associated with lower serum total cholesterol, LDL-C and apoB, whereas aggressive tumors showed increased LDLR and decreased HMGCR and CYP7B1. The detection rate and level of 27-HC increased with aggressiveness, reaching the highest frequency in PDTC/ATC [21]. CYP27A1 generates 27-HC and CYP7B1 clears it; thus, reduced CYP7B1 is consistent with 27-HC accumulation. LDL promoted proliferation and migration in thyroid cell models, CYP7B1 overexpression reduced CAL-62 proliferation and migration, and CYP27A1 knockdown blocked LDL-mediated proliferation [21].

These findings support a tumor-intrinsic uptake–conversion oxysterol model rather than a peripheral lipid-only model. LDLR, CYP27A1, CYP7B1 and 27-HC are candidate metabolic nodes in aggressive thyroid cancer, but current evidence does not justify inferring that systemic cholesterol lowering will necessarily reduce thyroid cancer risk or improve outcomes.

Cholesterol metabolism is also linked to targeted-therapy resistance. RTN3 was reported to have decreased in thyroid cancer and associated with disease progression, poor prognosis and MEK inhibitor insensitivity [22]. Mechanistically, RTN3 interacted with DHCR7 and promoted its ubiquitination; RTN3 loss stabilized DHCR7, increased cellular cholesterol and was linked to EGFR/ERK activation and tumor-progression phenotypes in experimental models. Simvastatin partially rescued this phenotype [22]. These data move cholesterol metabolism from an aggressiveness-associated observation toward a testable candidate mechanism of MEK inhibitor resistance. Clinical evidence for statin-based thyroid cancer intervention remains insufficient.

Systemic dyslipidemia and apolipoprotein studies provide related but distinct observations [23,24,25,26,27,28,29]. They do not directly report activity of the tumor-intrinsic LDLR-CYP27A1-CYP7B1 or RTN3-DHCR7 pathways and are evaluated separately below.

Across PTC, fatty acid uptake, synthesis, desaturation and oxidation are best viewed as a flexible network rather than a linear sequence. The cholesterol findings extend this principle into aggressive disease and targeted-drug adaptation. This network-level view predicts metabolic compensation and also raises the next question: which tumor, immune and spatial contexts determine whether a lipid program promotes recurrence or treatment resistance?

## 5. Immune-Spatial Lipid Niches and Therapy Resistance

Evidence at the tumor-microenvironment interface is emerging and mechanistically heterogeneous. Multi-omics and spatial studies identify lipid-associated immune states, while functional studies implicate APOE, CD36-associated macrophage programs and ferroptosis defenses. These findings are most useful when interpreted by histotype and treatment context rather than combined into a single universal immunometabolic pathway.

### 5.1. Lipid Metabolism, Immune Microenvironment and Recurrence Risk Subtyping

Lipid reprogramming also intersects with the immune microenvironment. Traditional recurrence risk stratification is based largely on clinicopathologic features, but multi-omics data indicate that PTCs within similar clinical risk categories can differ substantially in metabolic and immune states [1]. Four multi-omics subtypes were identified: low-risk/BRAF-like, high-risk/metabolic, high-risk/immune and high-risk/BRAF-like. The high-risk metabolic subtype had the poorest prognosis, supporting the concept of a metabolically defined recurrence risk state [1].

Multi-omics network analysis also suggested crosstalk between lipid metabolism and immune state. Lipid classes such as phosphatidylcholine, phosphatidylethanolamine and sphingomyelin formed important modules, while free fatty acids, diacylglycerols, phosphatidylglycerols and FABP5 appeared in network layers linking metabolites, genes, proteins and phosphoproteins with antigen-processing and presentation proteins [1]. Thus, fatty acid metabolism may not only reflect tumor cell energy and membrane requirements but may also interact with or report the tumor immune microenvironment.

CD36-positive macrophage studies further strengthen this immunometabolic theme. CD36-positive proinflammatory macrophages have been identified in the PTC microenvironment and linked to adverse outcome and recurrence risk [30]. These macrophages may interact with metabolically active ZCCHC12-positive tumor cells and support proliferation-related signaling through SPP1/PI3K-AKT. Although this evidence should be interpreted with attention to study design and validation, CD36 is biologically plausible as a fatty acid uptake and lipid-handling molecule that connects macrophage metabolism, inflammatory state and tumor cell behavior.

APOE provides a second, experimentally tractable link between tumor lipid biology and macrophage state. In PTC tissues and cell models, APOE expression accompanied higher xCT/SLC7A11, GPX4 and FTH1 and lower Fe^2+^ accumulation [29]. APOE knockdown increased Fe^2+^, MDA, ROS and ferroptosis-associated mitochondrial changes, whereas APOE overexpression produced the opposite pattern. DFO and PI3K inhibition partially reversed these effects, and THP-1-derived macrophage co-culture linked APOE-associated ferroptosis suppression to an Arg-1-high, IL-1beta-low M2-like state and increased PTC-cell migration and invasion. This is a coherent in vitro mechanism, but it remains limited by the absence of an in vivo tumor model, reliance on a simplified M1/M2 marker framework and the incomplete definition of how APOE controls iron handling.

Spatial omics studies are beginning to localize these interactions. Integrated spatial metabolomics and transcriptomics of PTC primary tumors, adjacent tissues and lymph node metastases mapped spatial metabolic–transcriptional landscapes and identified features associated with metastatic evolution [31]. These data suggest that PTC progression cannot be fully explained by bulk tissue averages. For lipid metabolism, future models should integrate lipid enzymes, lipid species and the spatial organization of immune cells and tumor cells.

### 5.2. Lipid Remodeling, Ferroptosis and Resistance Across DTC and ATC

Treatment-resistance evidence differs by histotype. In DTC, the principal questions are loss of iodine responsiveness and adaptation to MAPK pathway inhibition. In ATC, rapid growth, dedifferentiation and resistance to radiotherapy or chemotherapy place greater emphasis on membrane remodeling and ferroptosis escape. The ATC evidence base is smaller than that for PTC, but its functional focus makes it particularly relevant to sensitization strategies.

ATC is characterized by rapid progression, local invasion, distant metastasis and treatment resistance. Compared with PTC, the evidence base for lipid metabolism in ATC is smaller, but available studies suggest roles for lipogenesis, membrane remodeling and ferroptosis escape. Early work identified aberrant lipid metabolism in ATC and proposed SCD1 as a therapeutic target [19]. SCD1 may help ATC cells maintain membrane lipid homeostasis and avoid excessive lipid peroxidation during rapid growth and therapeutic stress.

More direct evidence connects lipid metabolism with radioresistance. YTHDF2-mediated stabilization of SREBF1 was reported to support lipid metabolic reprogramming and ferroptosis-associated radioresistance in ATC [7]. This mechanism is conceptually consistent with the METTL16/YTHDC2/SCD1 findings in PTC: m6A-associated regulators can control SREBP/SCD1-type lipid synthesis programs, thereby affecting lipid peroxidation and treatment response. However, different thyroid cancer subtypes, RNA targets and reader proteins may produce different biological consequences, so these pathways require subtype-specific validation.

Ferroptosis is a regulated cell death process driven by iron-dependent lipid peroxidation. Because membrane lipid composition and lipid peroxidation determine ferroptosis sensitivity, lipid metabolic reprogramming and ferroptosis are intrinsically connected. Reviews of ferroptosis in thyroid cancer emphasize SLC7A11, GPX4, glutathione metabolism, lipid peroxidation and iron metabolism as candidate therapeutic nodes [32,33]. In differentiated thyroid cancer cells, iodine-131 induced ferroptosis-like lipid peroxidation through SLC7A11 suppression and synergized with sulfasalazine [34]. In radioiodine-refractory DTC, metabolomic profiling distinguished RAIR from non-RAIR disease and implicated acetoacetate and ketone metabolism in iodine-uptake-related pathways [35].

ATC models further support metabolic and ferroptosis-directed sensitization strategies. Isoliquiritigenin was reported to suppress fatty acid synthesis and migration in ATC through AMPK/SREBF1 signaling [36]. Isobavachalcone plus doxorubicin suppressed ATC progression through ferroptosis activation [37], ultrasound-targeted microbubble-destruction-enhanced RSL3-induced ferroptosis [38], and GPX4-inhibition-induced ferroptosis and mTOR pathway suppression in thyroid cancer [39]. These studies suggest that the lipid synthesis and lipid peroxidation defense systems, especially the SREBF1/SCD1 and SLC7A11-GSH-GPX4 axes, may represent metabolic vulnerabilities for treatment-resistant thyroid cancer.

Lipid-associated immune niches and ferroptosis defenses connect the tumor-intrinsic programs described above to recurrence and therapy response. Their spatial and histotype specificity also limits simple extrapolation: a CD36-positive macrophage state in PTC is not equivalent to an SREBF1/SCD1-dependent radioresistant state in ATC. This distinction is essential to note before interpreting blood biomarkers or selecting treatment combinations.

## 6. Systemic Lipid Biomarkers and Clinical Interpretation

Circulating lipids are clinically accessible, but their evidence is predominantly associative. This section therefore separates diagnostic or prognostic performance from causal inference and repeatedly asks whether a blood marker adds information beyond established clinical variables or directly reflects an intratumoral lipid pathway.

### Blood Lipids, Lipoproteins and Risk or Metastasis Biomarkers

Peripheral blood lipid studies are close to clinical practice but are easy to overinterpret. Dyslipidemia, apolipoproteins and inflammatory–lipid composite markers may support risk assessment or metastasis prediction, but they do not directly measure intratumoral lipid flux. In a Chinese single-center retrospective study of 1650 patients with PTC and 882 controls with low-risk benign nodules, higher triglycerides and lower HDL-C were independently associated with prevalent PTC in both sexes [40]. The design cannot establish temporality, and the selection of a nodule-based control group, single-time-point measurements and residual metabolic or hormonal confounding limit causal interpretation.

The prospective AMORIS cohort provides a different level of evidence. Among 561,388 Swedish participants followed for more than 30 years, each standard deviation increase in total cholesterol and HDL-C was associated with modestly lower thyroid cancer risk, with hazard ratios of 0.91 and 0.86, respectively; LDL-C, triglycerides, ApoA-I and ApoB were not significantly associated [41]. Cases had lower total cholesterol and HDL-C decades before diagnosis, but several lipid and apolipoprotein markers declined further during the final 10 years before diagnosis. This pattern argues against treating measurements near diagnosis as simple etiologic exposures and suggests that both long-term host phenotype and reverse causation may contribute. The cohort nevertheless lacked histologic subtype, BMI and TSH data, and the effect sizes were small.

Mendelian randomization adds an exploratory causal inference layer, but the available DTC analysis requires particular caution [42]. Univariate models reported associations for total cholesterol, HDL-C, ApoB and the ApoB/ApoA-I ratio, whereas none remained significant when the four correlated traits were entered together in multivariable MR. The DTC outcome GWAS was small, the article reported inconsistent case–control counts between sections, and some effect estimates were extreme with wide confidence intervals. These results are better treated as hypothesis-generating genetic signals than as proof that modifying a circulating lipid trait will alter DTC risk.

Composite inflammatory–lipid markers are also of interest. In a single-center study that retained 82 of 200 screened patients, MHR was associated with central lymph node metastasis after propensity-score matching (OR 1.76, 95% CI 1.20–2.88) [43]. Integrated public scRNA-seq data from 17 tissues identified cholesterol-homeostasis-associated tumor states and a CD68-positive C3 macrophage cluster linked to metastatic features, while co-culture experiments supported a migration-promoting M2-like macrophage phenotype. This multimodal triangulation is biologically informative, but it does not show that blood MHR directly measures the intratumoral mechanism, and the clinical estimate lacks external validation. A final literature review identified a separate retrospective propensity-score-matched cohort in which the platelet-to-HDL-C ratio was independently associated with lymph node metastasis and contributed to a moderate-discrimination nomogram [44]. Both indices remain candidate biomarkers whose value depends on external validation and incremental performance beyond ultrasound and clinicopathologic features.

Metabolomic and lipidomic studies also support diagnostic or metastatic biomarker development. A serum-free fatty acid panel including C16:1, C18:2, C20:4 and C22:6 distinguished benign thyroid disease from thyroid cancer in 664 serum samples [14]. Plasma-combined metabolomics and lipidomics identified 113 differential metabolites and 236 differential lipids between PTC and healthy controls [15]. These biomarker studies do not replace mechanistic tissue studies, but they provide a translational route toward noninvasive diagnosis, risk stratification and metabolic phenotyping.

Metabolomics and lipidomics provide an additional biomarker route. Studies have used metabolomics to predict lymph node metastasis [45], plasma metabolomics/lipidomics to identify PTC diagnostic candidates [15], salivary metabolomics to detect metastatic PTC after surgery [46], fine-needle aspiration metabolomics to distinguish thyroid nodules [47], and NMR-based tissue or plasma profiling to identify papillary thyroid microcarcinoma [48]. These approaches are attractive because they can be aligned with clinical pathways, but multicenter, blinded and prospective validation, including incremental value over ultrasound, cytology, pathology and clinicopathologic risk features, is required before clinical recommendation.

Blood lipids, lipoproteins and metabolite panels may support noninvasive phenotyping, but their associations cannot be used as surrogates for tumor lipid dependency. The most informative future designs will pair serial blood measurements with tumor lipidomics, enzyme expression and clinical outcomes. This paired strategy provides the clinical sampling framework needed to test the therapeutic hypotheses prioritized next.

## 7. Therapeutic Prioritization and Translational Roadmap

Candidate targets were prioritized by four criteria: directness to a defined thyroid cancer context, functional causality, availability of a measurable selection or pharmacodynamic biomarker, and the feasibility of a rational combination. On this basis, the field currently supports three tiers of development rather than an undifferentiated list of druggable nodes (Table 2). None is established for routine thyroid cancer care.

### 7.1. Tier 1: Context-Defined Sensitization Strategies

The highest-priority experiments are combination strategies anchored to a specific resistance state. These include FAO inhibition in BRAFV600E tumors that increase FAO during BRAF inhibition [10], suppression of RTN3-low/DHCR7-high cholesterol synthesis during MEK inhibition [22], and ferroptosis sensitization during radioiodine, radiotherapy or chemotherapy [7,34,37,38,39]. Validation should begin in molecularly annotated organoids and in vivo models with combination dose matrices, target-engagement assays and serial lipid or redox readouts. Key safety concerns are reduced energetic flexibility in heart, liver and immune cells for FAO-directed approaches; muscle and hepatic toxicity and drug interactions for statin-based combinations; and oxidative injury to normal tissues for systemic ferroptosis induction. Intermittent dosing, treatment sequencing and tumor-selective delivery should therefore be tested rather than assumed.

### 7.2. Tier 2: Tumor-Intrinsic Lipid Dependencies

SCD1, the PC-SREBP1c-FASN axis and combined LPL/FATP2-mediated uptake plus FASN-mediated synthesis are biologically compelling but require stronger replication or therapeutic window data [6,13,16,17,18,19]. SCD1 is the most advanced pharmacologic example because MTI-301 has entered a pan-solid-tumor phase I study, although no thyroid-specific results are available. Candidate selection markers include low METTL16 with high SCD1, high PC/FASN/SREBP1c, and joint LPL/FATP2/FASN activity. Safety surveillance should include tissues dependent on lipid homeostasis, particularly skin and ocular surface for SCD1 inhibition, and systemic metabolic effects for uptake or synthesis blockade.

Metabolic adaptation must be treated as a primary endpoint. Restricting exogenous lipid supply can select for SREBP1-FASN-ACC-dependent synthesis; blocking synthesis can increase lipid scavenging, storage lipid mobilization or CPT1A-dependent oxidation; and FAO inhibition can redirect fatty acids toward membrane synthesis or lipotoxic storage. Future studies should therefore quantify isotope-resolved flux under physiologic lipid conditions, test paired blockade only after documenting compensation, and include rescue experiments that distinguish pathway dependence from nonspecific cytotoxicity.

### 7.3. Tier 3: Immune Lipid and Systemic Interventions

CD36-positive macrophages, APOE-associated polarization and SPP1/PI3K-AKT interactions remain exploratory therapeutic hypotheses [29,30]. CD36 and related lipid-handling programs also maintain normal macrophage, platelet, cardiac and skeletal–muscle functions; systemic blockade could alter tissue repair or host lipid handling without selectively suppressing a tumor-promoting niche. Spatially resolved target expression, paired tumor and normal-tissue profiling, and state-selective delivery should precede immunotherapy combinations. Likewise, observational blood lipid associations do not justify empirical statins, ketogenic diets or other systemic metabolic interventions outside a trial.

Section synthesis: The near-term translational opportunity is biomarker-guided sensitization, not lipid lowering as a generic anticancer strategy. A credible development path requires a defined histotype and resistance context, a measurable metabolic state, evidence of target engagement, prospective monitoring of compensation and prespecified normal-tissue safety endpoints.

## 8. Limitations and Research Priorities

Several limitations should temper interpretation. Evidence remains uneven across histotypes. PTC has the strongest data for fatty acid metabolism, SCD1 regulation and recurrence risk multi-omics, whereas ATC evidence is smaller and concentrated on lipid remodeling, ferroptosis and therapy resistance. FTC, PDTC and MTC remain underrepresented, so findings should not be extrapolated across histologic subtypes without validation.

Study designs are also imbalanced. Multi-omics and bioinformatic studies identify candidate pathways and subtypes, but many findings remain associative. Functional experiments strengthen causal inference, yet sample sizes, model systems and in vivo validation are often limited. Clinical lipid studies are vulnerable to confounding, reverse causation and inconsistent phenotypes. The available Mendelian randomization analysis is exploratory, and inflammatory–lipid ratios lack independent external validation. Even the explicit 0–10 evidence score cannot eliminate author judgment or compensate for sparse replication.

The relationship between tumor-intrinsic lipid metabolism and systemic host metabolism remains unresolved. Peripheral LDL-C, triglycerides, HDL-C and apoB do not necessarily reflect intratumoral flux. Aggressive tumors may increase LDLR-mediated uptake, reduce circulating LDL-C or apoB and increase intratumoral 27-HC [21]. Prospective studies should therefore collect serial blood lipids, tumor lipidomics, metabolic enzyme expression and outcomes in parallel.

This review also has methodological limitations. Evidence identification remained PubMed-centered despite supplementary searches of Wanfang Data, VIP/CQVIP and public trial and omics repositories. Embase, Web of Science, Scopus, CNKI and SinoMed were not searched successfully. Screening and evidence appraisal were undertaken for a narrative synthesis rather than by two independent reviewers, and no design-specific risk-of-bias assessment was performed. The reported counts are an audit trail, not a PRISMA study-selection flow.

Database, language and publication bias toward positive mechanistic findings remain possible. Some conclusions rely on limited thyroid cancer cell line panels whose driver mutations, lineage state, culture lipids and oxygen conditions may alter metabolic dependence and drug response. Target-specific toxicity and adaptive rewiring are incompletely characterized, and the synthesis may favor vulnerabilities with clearer translational narratives.

Priority studies should use subtype- and driver-stratified cohorts; paired blood and tumor lipidomics; isotope-resolved flux; spatial transcriptomic, proteomic and lipidomic mapping; and functional perturbation in organoids and in vivo models. Therapeutic studies should prespecify selection biomarkers, target-engagement measures, adaptive-flux endpoints, normal-tissue controls and treatment sequence. A future prospectively registered systematic review should add Embase, Web of Science, Scopus, CNKI and SinoMed; conduct backward and forward citation chasing; use dual independent screening and extraction; appraise risk of bias by study design; and assess eligible non-English full texts.

## 9. Conclusions and Future Directions

Lipid metabolic reprogramming is histotype- and context-dependent across tumor cells, host metabolism, immune-spatial niches and treatment response. PTC evidence supports altered fatty acid acquisition, synthesis, desaturation, oxidation and membrane remodeling linked to recurrence and metastasis. In aggressive tumors, cholesterol synthesis, FAO adaptation and ferroptosis defense are plausible resistance mechanisms. The near-term strategy is biomarker-guided sensitization in defined contexts, not empirical lipid lowering. Translation requires independent replication, paired blood–tumor studies, spatial and functional validation, compensation testing and prospective trials with pharmacodynamic and safety endpoints.

Table 1 uses a five-domain score (D, H, C, M and R; 0–2 points each; total 0–10) defined in the Section 2 (Review Approach) and Supplementary File S1. Scores of 0–3 dictate categorization as emerging and scores of 4–6 dictate categorization as moderate; ‘preclinical’ denotes functional evidence without clinical validation. Moderate–high preclinical requires 7–10 points plus direct thyroid evidence, functional perturbation, model depth and independent support. Single-study chains are capped at moderate preclinical categorization. The independent-study count includes distinct primary publications directly supporting the broad chain, not reviews or repeated analyses of the same dataset.

**Table 1:** Evidence chains in thyroid cancer lipid metabolism.

Evidence Chain	Main Context	Key Nodes	Evidence Base	Independent Studies (*n*)	Score and Category	Clinical Maturity	Representative Evidence	Translational Implication
Fatty acid hydrolysis, uptake and oxidation	PTC progression and prognosis	LPL, FATP2/SLC27A2, CPT1A	Multi-omics, tissue validation and migration assays	3	D2/H1/C1/M0/R1 = 5; moderate	Biomarker and preclinical hypothesis	Storage lipids decrease, membrane lipids increase, and uptake/oxidation nodes are associated with progression [6,8,11]	Test uptake and FAO dependencies with flux and in vivo validation
Metabolic recurrence risk subtype	PTC recurrence stratification	TG, FFA, FABP5, lipid modules	Multi-omics in 102 patients	1	D2/H2/C0/M0/R0 = 4; moderate	Stratification hypothesis	A high-risk metabolic subtype was associated with worse prognosis [1]	Requires external, prospective validation
De novo lipogenesis	PTC invasion and growth	PC, AKT/mTOR, SREBP1c, FASN, ACC1, ACLY	Patient samples, cells, xenografts and rescue	2	D2/H1/C2/M2/R0 = 7; moderate preclinical	Preclinical target hypothesis	PC perturbation altered lipid accumulation, invasion and xenograft growth; complementary ACC1 evidence exists [12,16]	Deep multilevel evidence, but the specific PC chain lacks independent replication
SCD1-dependent desaturation	PTC and ATC malignant phenotypes	METTL16, YTHDC2, SCD1	Tissues, multi-omics, perturbation and in vivo models	4	D2/H1/C2/M2/R1 = 8; moderate–high preclinical	Preclinical target hypothesis	Independent studies support SCD1-related programs across PTC and ATC [17,18,19,20]	Biomarker-selected SCD1 testing is justified preclinically
Cholesterol and oxysterol metabolism	High-risk PTC and PDTC/ATC	LDLR, CYP27A1, CYP7B1, 27-HC	Patient lipids, tumor 27-HC and cell function studies	1	D2/H1/C2/M1/R0 = 6; moderate preclinical	Mechanistic and stratification hypothesis	Aggressive tumors showed greater LDL uptake and 27-HC accumulation [21]	Separate systemic cholesterol from tumor uptake–conversion biology
Cholesterol synthesis and targeted resistance	MEK inhibitor insensitivity	RTN3, DHCR7, cholesterol, EGFR/ERK	TCGA, interaction, cell and animal studies	1	D2/H1/C2/M2/R0 = 7; moderate preclinical	Preclinical sensitization hypothesis	RTN3 loss stabilized DHCR7 and increased cholesterol; simvastatin partially rescued the phenotype [22]	Prioritize RTN3-low/DHCR7-high models for replication
Lipid–immune interface	PTC recurrence and spatial heterogeneity	CD36+ macrophages, APOE, SPP1/PI3K-AKT	Multi-omics, spatial and mechanistic studies	3	D1/H1/C1/M0/R0 = 3; emerging	Exploratory biomarker hypothesis	Distinct studies link lipid-associated macrophage states and spatial heterogeneity to PTC progression [29,30,31]	Mechanisms require spatially matched functional replication
Ferroptosis and radioiodine sensitization	DTC and RAIR-DTC	SLC7A11, GPX4, GSH, lipid peroxidation	DTC cells and one in vivo model	1	D2/H0/C1/M1/R0 = 4; moderate preclinical	Preclinical sensitization hypothesis	I-131 induced ferroptosis and synergized with sulfasalazine in DTC models [34]; the RAIR-DTC acetoacetate/NIS study is contextual rather than direct ferroptosis evidence [35]	Replicate independently in organoids/in vivo with normal thyroid controls and dosimetry
Ferroptosis and ATC treatment resistance	ATC radiation and chemotherapy resistance	YTHDF2/SREBF1/SCD1, GPX4, SLC7A11	ATC cells and preclinical models	4	D2/H0/C2/M1/R1 = 6; moderate preclinical	Preclinical sensitization hypothesis	Multiple studies support lipid-remodeling or ferroptosis-inducing strategies [7,37,38,39]	Replicate with molecular selection and normal-tissue safety models

Note: The PC-AKT/mTOR-SREBP1c-FASN chain was conservatively classified as moderate preclinical because its most detailed causal support comes from one principal study [16], despite complementary ACC1 evidence [12]. Independent replication in larger, molecularly annotated cohorts remains necessary.

**Table 2:** Candidate therapeutic targets and combination strategies in thyroid cancer lipid metabolism.

Tier	Direction and Candidate	Biomarker-Defined Context	Proposed Application	Evidence/Maturity	Potential Toxicity	Expected Adaptation	Next Validation Step
1	Suppress FAO; CPT1A/FAO-directed strategy	BRAFV600E, therapy-induced FAO/PGC1alpha state	Combine with BRAF inhibition	Preclinical and organoid-supported [10]	Cardiac, hepatic and immune energetic stress	Greater lipid uptake or synthesis; fatty acid storage	Annotated organoids and in vivo dose-sequence studies with flux and H3K9ac readouts
1	Suppress cholesterol synthesis; DHCR7/HMGCR	RTN3-low/DHCR7-high, MEK-insensitive tumors	Combine with MEK inhibition	Single-study preclinical rationale [22]	Muscle/hepatic effects and drug interactions	LDLR-mediated uptake or alternative sterol synthesis	Independent replication, target engagement and combination tolerability
1	Induce ferroptosis; SLC7A11/GPX4	RAI-responsive DTC, RAIR-DTC or ferroptosis-defended ATC	Sensitize RAI, radiotherapy or chemotherapy	Preclinical [7,34,37,38,39]	Normal-tissue oxidative injury	Antioxidant, iron-handling and lipid-remodeling escape	Organoid/in vivo efficacy, normal thyroid controls and sequence optimization
2	Target SCD1 or upstream m6A-SCD1 control	METTL16-low/SCD1-high PTC; SCD1-dependent ATC	SCD1 inhibition alone or with standard therapy	Thyroid preclinical; pan-solid-tumor phase I active	Skin/ocular lipid-homeostasis effects; monitored neurologic safety	Exogenous MUFA uptake, FAO or alternative lipogenesis	Prospective biomarker assay and thyroid-specific pharmacodynamic cohort
2	Inhibit PC-SREBP1c lipogenesis	PC/FASN/SREBP1c-high PTC	Block synthesis or combine after documented uptake compensation	Moderate preclinical [12,16]	Systemic metabolic and hepatic effects	Lipid scavenging, droplet mobilization and FAO	Independent replication, isotope tracing and rescue-based combination tests
2	Block lipid supply; LPL/FATP2 plus FASN	Joint uptake- and synthesis-dependent PTC	Dual supply blockade	Preclinical [6,13]	Normal-tissue lipid transport effects	Alternative transporters and stored-lipid mobilization	Physiologic-lipid culture, in vivo window and adaptive-flux profiling
3	Modulate immune lipid handling; CD36/APOE/SPP1	Spatially defined macrophage–tumor niches	State-selective or locally delivered immune metabolic therapy	Emerging hypothesis [29,30,31]	Macrophage, platelet, cardiac and muscle homeostasis	Myeloid-state replacement or alternate scavenger pathways	Spatial target confirmation and paired tumor/normal functional models
3	Systemic interventions; statins or dietary strategies	No validated thyroid-specific selection marker	Trial-only exploratory intervention	Not clinically established	Class-specific metabolic effects and interactions	Host compensation; uncertain intratumoral exposure	Prospective biomarker-defined trials; no empirical routine use

## Figures and Tables

**Figure 1:**
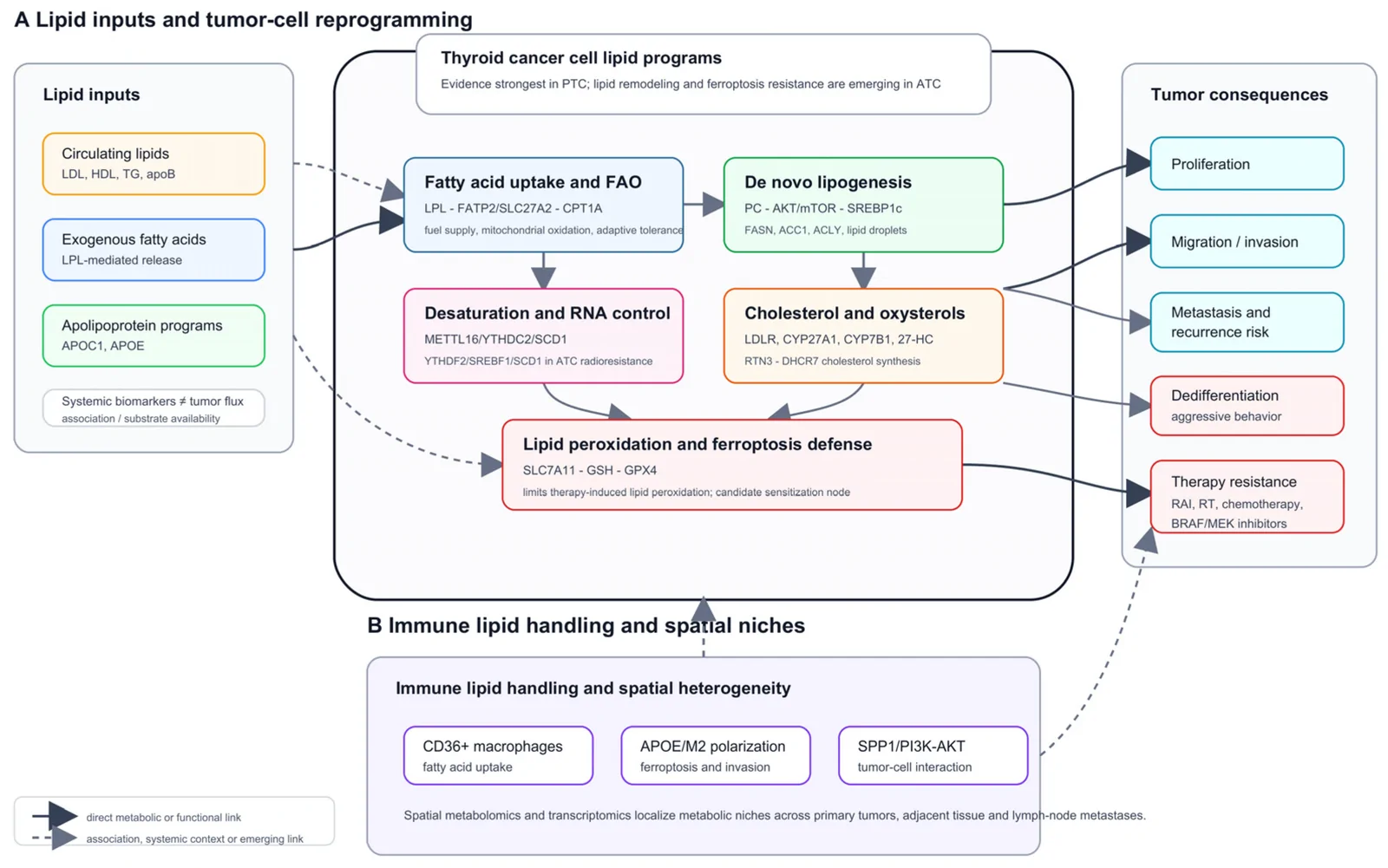
Integrated lipid metabolic reprogramming.

**Figure 2:**
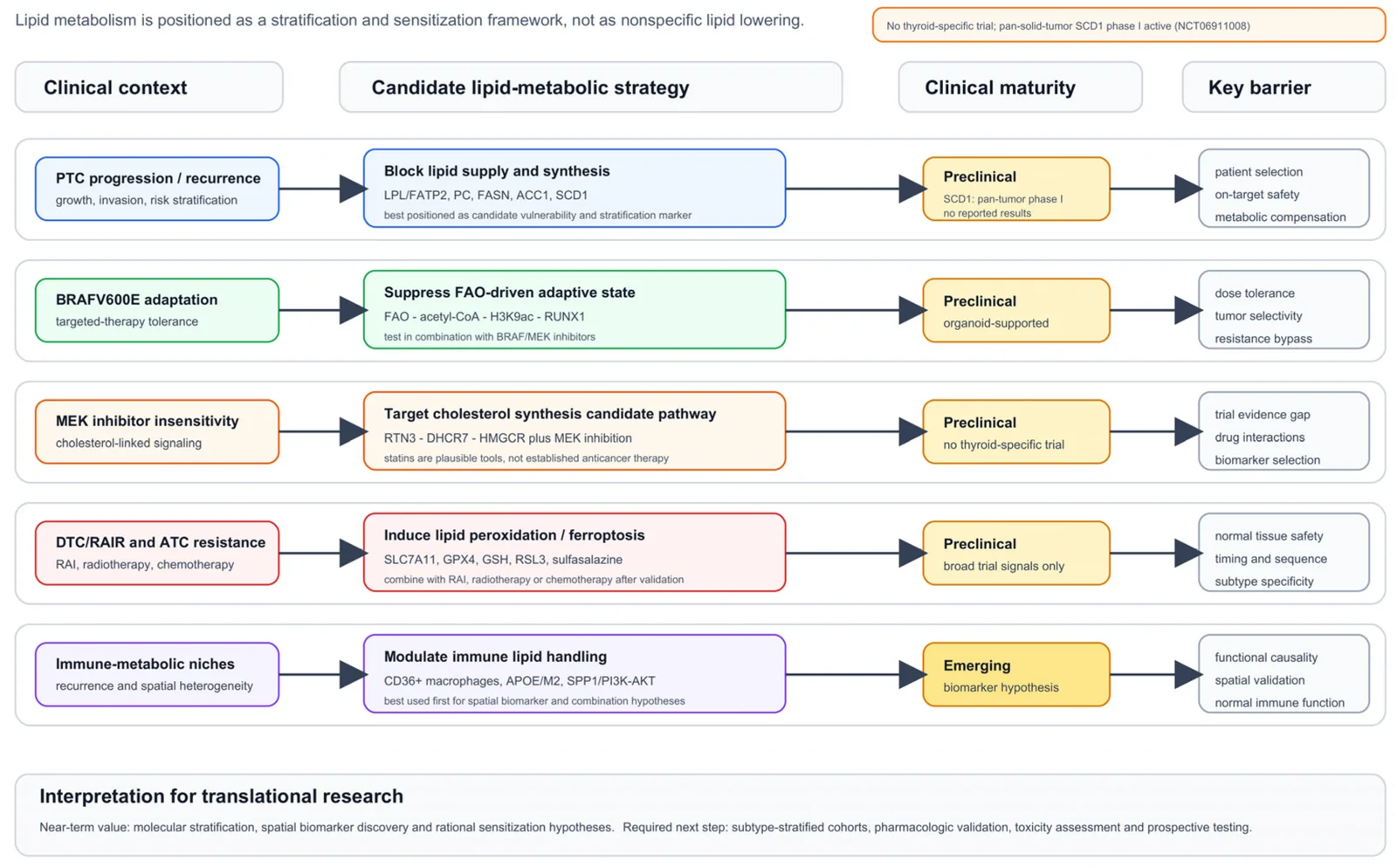
Translational opportunities for targeting lipid metabolism.

## Data Availability

No new data were created or analyzed in this study. Data sharing is not applicable to this article.

## Supplementary Materials

The following supporting information can be downloaded at: https://www.mdpi.com/article/10.3390/metabo16100736/s1, Supplementary File S1: Search strategy and evidence screening, including full PubMed search strings, expanded evidence lane summaries, screening accounting, the narrative evidence appraisal rubric, targeted PubMed refresh results, public database scan results and audit trail file notes.

## Abbreviations

27-HC: 27-hydroxycholesterol; ACC: acetyl-CoA carboxylase; ACLY: ATP citrate lyase; APO: apolipoprotein; ATC: anaplastic thyroid carcinoma; BRAFi: BRAF inhibitor; CPT1A: carnitine palmitoyltransferase 1A; DTC: differentiated thyroid carcinoma; FAO: fatty acid oxidation; FASN: fatty acid synthase; FATP2: fatty acid transport protein 2; FTC: follicular thyroid carcinoma; GPX4: glutathione peroxidase 4; HDL-C: high-density lipoprotein cholesterol; HMGCR: 3-hydroxy-3-methylglutaryl-CoA reductase; LDL-C: low-density lipoprotein cholesterol; LDLR: low-density lipoprotein receptor; LNM: lymph node metastasis; LPL: lipoprotein lipase; MHR: monocyte-to-HDL cholesterol ratio; MTC: medullary thyroid carcinoma; NIS: sodium iodide symporter; PC: pyruvate carboxylase; PDTC: poorly differentiated thyroid carcinoma; PTC: papillary thyroid carcinoma; RAI: radioactive iodine; RAIR: radioiodine-refractory; SCD1: stearoyl-CoA desaturase 1; SLC7A11: solute carrier family 7 member 11; TG: triglyceride.
