# Supplementary material for: Lipid Metabolic Reprogramming in Thyroid Cancer: Systemic Biomarkers, Tumor Dependencies and Therapeutic Resistance
Source: Metabolites. 2026 Sep 30;16(10):736. doi: 10.3390/metabo16100736 (PMC13707171; doi:10.3390/metabo16100736)
Supplement: Supplementary file 1 [file metabolites-16-00736-s001.zip › metabolites-4516644-supplementary.pdf]

Supplementary File S1. Search strategy and evidence screening

Manuscript: Lipid metabolic reprogramming in thyroid cancer: systemic biomarkers, tumor dependencies and therapeutic resistance

Search dates: 25 May 2026 for PubMed; 26 May 2026 for supplementary public-database scans; targeted PubMed refresh on 5 June 2026; supplementary Wanfang Data and VIP/CQVIP searches on 7 June 2026; ClinicalTrials.gov/NCI trial-status refresh on 12 June 2026.

Review type: mechanism-oriented narrative review. This review was not designed as a PRISMA systematic review or meta-analysis. The search documentation is provided to improve reproducibility and transparency, but the screening was not performed as formal dual independent systematic-review screening and no inter-rater agreement statistic was calculated.

PubMed search approach

PubMed searches were performed without date or article-type restrictions. The search strategy combined thyroid cancer terms with lipid metabolism, lipidomics/metabolomics, cholesterol/lipoprotein, fatty acid metabolism, ferroptosis/lipid peroxidation and immune microenvironment terms.

Initial broad PubMed search

```
("Thyroid Neoplasms"[Mesh] OR "thyroid cancer"[Title/Abstract] OR "thyroid
carcinoma"[Title/Abstract] OR "thyroid neoplasm*"[Title/Abstract] OR "papillary thyroid
carcinoma"[Title/Abstract] OR PTC[Title/Abstract] OR "follicular thyroid
carcinoma"[Title/Abstract] OR FTC[Title/Abstract] OR "medullary thyroid
carcinoma"[Title/Abstract] OR MTC[Title/Abstract] OR "anaplastic thyroid
carcinoma"[Title/Abstract] OR ATC[Title/Abstract] OR "differentiated thyroid
cancer"[Title/Abstract])

AND

("Lipid Metabolism"[Mesh] OR "Fatty Acid Metabolism"[Mesh] OR "Cholesterol Metabolism"[Mesh] OR
lipid*[Title/Abstract] OR lipidomic*[Title/Abstract] OR metabolomic*[Title/Abstract] OR "fatty
acid"[Title/Abstract] OR lipogenesis[Title/Abstract] OR "fatty acid oxidation"[Title/Abstract] OR
"cholesterol metabolism"[Title/Abstract] OR "phospholipid"[Title/Abstract] OR
```

```
"sphingolipid"[Title/Abstract] OR "lipid droplet"[Title/Abstract] OR
dyslipidemia[Title/Abstract])
```

Result: 30 records.

Targeted lipid-gene PubMed search

The targeted search combined thyroid cancer terms with lipid-related genes and pathways, including FASN, SREBF1/SREBP1, SCD/SCD1, CPT1A, CD36, FABP, ACSL, HMGCR, SREBF2/SREBP2, PPAR, LXR/NR1H, mTOR, AMPK, PI3K/AKT, BRAF V600E and TERT promoter.

Result: 50 records.

Expanded PubMed evidence lanes

The expanded PubMed search was organized into six overlapping evidence lanes. After deduplication across lanes, 1,478 unique records were retained. A priority shortlist of 150 records was generated for title/abstract screening.

Evidence lane	Query summary	Records
Broad lipid metabolism	Thyroid cancer terms AND lipid, fatty acid, cholesterol, lipoprotein, dyslipidemia, triglyceride, phospholipid, sphingolipid, ceramide, lipid droplet, ferroptosis, lipid peroxidation, lipidomics or metabolomics terms	943
Lipidomics/metabolomics	Thyroid cancer terms AND lipidomics, metabolomics, metabolome, mass spectrometry or LC-MS terms	534
Cholesterol/lipoproteins/dyslipidemia	Thyroid cancer terms AND cholesterol, lipoprotein, dyslipidemia, LDL, HDL, triglyceride, apolipoprotein or statin terms	389

Evidence lane	Query summary	Records
Fatty acid/lipogenesis targets	Thyroid cancer terms AND fatty acid, lipogenesis, FASN, SCD1, CPT1A, FABP, CD36, SREBF1, SREBP1, ACLY, ACC1 or pyruvate carboxylase terms	111
Ferroptosis/lipid peroxidation	Thyroid cancer terms AND ferroptosis, lipid peroxidation, GPX4, ACSL4, SLC7A11 or iron death terms	150
Immune lipid microenvironment	Thyroid cancer terms AND CD36, macrophage, tumor microenvironment or immune terms AND lipid, fatty acid, cholesterol or metabolism terms	154

Screening and prioritization

The 150-record priority shortlist was screened by title and abstract. Studies were prioritized when they met one or more of the following criteria:

- Direct relevance to thyroid cancer or a defined thyroid cancer subtype.
- Direct assessment of lipid metabolism, lipidomics/metabolomics, cholesterol/lipoprotein biology, fatty acid uptake/synthesis/oxidation, lipid droplets, ferroptosis/lipid peroxidation or immune lipid handling.
- Use of patient tissue, blood-based cohorts, spatial omics or clinically annotated samples.
- Mechanistic perturbation by knockdown, overexpression, pharmacologic inhibition, rescue experiments, animal models or patient-derived organoids.
- Relevance to recurrence, lymph-node metastasis, dedifferentiation, radioiodine resistance, radiotherapy resistance, chemotherapy resistance or targeted-therapy adaptation.

Studies were deprioritized when they were not thyroid-cancer-specific, had only indirect metabolic relevance, were purely prognostic-signature studies without functional or clinical validation, or were prior reviews used only for background.

Priority-screen accounting

The 150 screened priority records were assigned to the following narrative-review categories. These categories were used to guide evidence weighting and were not intended as a PRISMA inclusion flow.

Screening category	Records	Interpretation
Core-synthesis priority records	85	Records prioritized for direct thyroid-cancer lipid-metabolism evidence with patient samples, mechanistic experiments, omics data, clinical cohorts or treatment-resistance relevance; full-text availability and local archiving varied
Secondary inclusion evidence	28	Relevant supportive evidence, often requiring lower weighting because of narrower mechanism, indirect lipid connection or limited validation
Background or hypothesis-generating evidence	21	Reviews, public-database/prognostic-signature studies or broader context papers used for framing rather than strong causal inference
Excluded	16	Retracted articles, non-thyroid-cancer lipid-metabolism records, peripheral clinical questions or records outside the review scope

The final manuscript cited 48 references selected from the priority, background, Chinese-language and final-refresh evidence pools. Cited studies were chosen to represent the strongest available evidence chains while limiting overreliance on prognostic-signature-only, review-level or non-thyroid-cancer-specific records.

Flow-style screening summary

Stage	Records	Notes
Expanded PubMed evidence lanes	2,181 lane-level records	Records were counted across six overlapping lanes before deduplication
Deduplicated candidate pool	1,478	Unique records retained after deduplication across lanes
Priority title/abstract screen	150	Records prioritized for thyroid cancer specificity, lipid-metabolism relevance, clinical annotation, omics evidence or functional validation
Core-synthesis priority records	85	Records prioritized for detailed review; not all records were locally archived as full text
Secondary inclusion evidence	28	Supportive evidence with narrower mechanism, indirect lipid connection or limited validation
Background/hypothesis-generating evidence	21	Reviews, public-database/prognostic-signature studies or broader context papers
Excluded after priority screening	16	Non-thyroid-cancer records, peripheral clinical questions, retracted or out-of-scope records
Cited in final manuscript	48	Selected to represent the strongest and most relevant evidence chains after targeted refresh and Chinese-language supplementary searching

Evidence appraisal rubric

Evidence strength was assigned with an author-designed, five-domain narrative score. Each evidence chain received 0-2 points for thyroid/histotype directness (D), human relevance (H), functional causality (C), model depth (M) and independent replication of the same mechanism (R), for a total of 0-10. A score of 0 indicates absent or indirect support, 1 partial support and 2 strong support within each domain. This framework is not formal GRADE because the evidence base is dominated by omics, observational biomarker and preclinical mechanistic studies rather than comparative intervention trials. Distinct primary publications directly supporting each broad chain were also counted; reviews and repeated analyses of the same dataset were excluded.

Component or category	Operational definition
Directness (D), 0-2	0: non-thyroid or indirect; 1: thyroid cancer but mixed/uncertain histotype or correlative target assignment; 2: direct evidence in a defined thyroid cancer histotype and pathway.
Human relevance (H), 0-2	0: no human material; 1: patient tissue, blood or public human omics; 2: clinically annotated multi-cohort, prospective or externally validated human evidence.
Functional causality (C), 0-2	0: association only; 1: single genetic or pharmacologic perturbation; 2: orthogonal perturbation and rescue or convergent target-specific evidence.
Model depth (M), 0-2	0: descriptive data or one cell context; 1: multiple cell models, organoid or one in vivo model; 2: convergent in vivo plus organoid/PDX or comparably deep models.
Independent replication (R), 0-2	0: one study/dataset; 1: independent study or external cohort supporting the same mechanism; 2: repeated independent validation across cohorts or model systems.
Emerging (0-3)	Early, mainly associative or single-context evidence. 'Emerging preclinical' indicates that functional perturbation is present but the total score remains 0-3.
Moderate (4-6)	Direct human or multi-omics support, limited causal evidence or partial replication. 'Moderate preclinical' indicates functional evidence without prospective clinical validation.
Moderate-high preclinical (7-10)	Requires direct thyroid evidence, functional perturbation, model depth and independent support. A mechanistically deep single study is capped at Moderate preclinical.
Clinically established	Prospective thyroid-cancer-specific intervention or biomarker-validation evidence sufficient to support clinical use; no evidence chain reached this category.
Independent-study count	Number of distinct primary publications directly supporting the broad evidence chain. Reviews, background sources and repeated analyses of the same dataset are excluded.

Targeted PubMed refresh

A targeted PubMed refresh was performed on 5 June 2026 for records published from 25 May 2026 to 5 June 2026. The refresh queried thyroid cancer terms combined with lipid metabolism, fatty acid metabolism, cholesterol, lipidomics/metabolomics, ferroptosis/lipid peroxidation, SCD1, SREBF1, CD36, CPT1A, FASN, GPX4 and SLC7A11.

Refresh result	Interpretation
Broad thyroid cancer/lipid query: 4 records	One newly identified retrospective PTC platelet-to-HDL-C ratio study was incorporated as supportive biomarker evidence; other records were already captured in the prior expanded database or were methodologic/background rather than core mechanistic evidence
Cholesterol/oxysterol/statin query: 0 records	No new cholesterol, oxysterol or statin-related study requiring manuscript revision was identified
Ferroptosis/lipid-peroxidation query: 1 record	The record was a review on immunotherapy and ferroptosis regulation already present in the expanded candidate database; it was not used as primary mechanistic evidence

Refresh result	Interpretation
Fatty-acid/lipogenesis query: 0 records	No new fatty-acid uptake, oxidation, desaturation or lipogenesis study requiring manuscript revision was identified

Supplementary public-database scan

To reduce PubMed-only bias, a supplementary scan was performed on 26 May 2026.

Source	Search focus	Main interpretation
ClinicalTrials.gov and NCI trial registry	Thyroid cancer with lipid metabolism, statin, ketogenic diet, SCD1 inhibition or ferroptosis/iron-related terms	No thyroid cancer-specific lipid-metabolism-targeted anticancer trial was identified. A 12 June 2026 refresh identified an active first-in-human phase I study of the SCD1 inhibitor MTI-301 in refractory metastatic or unresectable solid tumors (NCT06911008); it is not thyroid-cancer-specific and has no reported safety or efficacy results
PRIDE	Thyroid cancer, thyroid carcinoma, papillary thyroid cancer lipid, anaplastic thyroid cancer	No directly matching proteomics project was identified through the initial API scan
BioStudies/ArrayExpress	Thyroid cancer lipidomics/metabolomics and thyroid carcinoma datasets	Returned mainly transcriptomic, miRNA, ChIP-seq or treatment-response datasets rather than direct lipidomics/metabolomics datasets
NCBI GEO/GDS	Thyroid cancer lipidomics/metabolomics and thyroid carcinoma lipid metabolism	Direct lipidomics/metabolomics returns were limited; GSE171966 was noted as a potentially relevant SREBF1-related transcriptomic dataset

Source	Search focus	Main interpretation
MetaboLights	Thyroid cancer metabolomics/lipidomics	Direct reusable thyroid-cancer lipidomics/metabolomics datasets were not readily identified in the preliminary scan

Supplementary Chinese-language database search

To reduce language bias, Wanfang Data and VIP/CQVIP were searched on 7 June 2026. These searches supplemented the PubMed-centered evidence review; they were not treated as a formal systematic-review update. Results were screened for direct thyroid-cancer and lipid-metabolism relevance, and database counts were not interpreted as eligible-study counts.

Wanfang Data

Query	Records displayed	Screening interpretation
甲状腺癌 脂质代谢	59	The most directly relevant database-unique journal record was a 2018 patient-tissue study that used array-based discovery followed by RT-PCR validation in 46 paired PTC and adjacent-tissue samples; SLC27A6/FATP6 and FABP3 mRNA were increased in tumor tissue
甲状腺癌 脂肪酸	50	Results included mechanistic theses, reviews and records overlapping the PubMed fatty-acid evidence pool
甲状腺癌 胆固醇	181	Many records addressed systemic lipids, thyroid-hormone withdrawal or indirect clinical associations rather than tumor-intrinsic cholesterol metabolism

Query	Records displayed	Screening interpretation
甲状腺癌 铁死亡	79	The 2026 timosaponin AIII/auranofin liposome study was already present in the PubMed candidate database and was not a database-unique discovery
甲状腺癌 脂质组学	195	Low-specificity result because the default interface split Chinese terms and retrieved false-positive uses such as liposome and tissue-group wording
甲状腺癌 代谢组学	83	Included reviews and primary metabolomics studies, many of which were already represented in the PubMed evidence pool
甲状腺癌 血脂	138	Many records concerned levothyroxine withdrawal before radioiodine treatment or systemic associations rather than tumor-intrinsic lipid flux

VIP/CQVIP

The public interface was searched using quoted concepts joined by `AND`. Platform-displayed totals and Chinese-language subsets were recorded because the interface also retrieved foreign-language records.

Query	All records	Chinese-language records
"甲状腺癌" AND "脂质代谢"	68	43
"甲状腺癌" AND "脂肪酸"	89	61
"甲状腺癌" AND "胆固醇"	175	141
"甲状腺癌" AND "铁死亡"	131	57
"甲状腺癌" AND "脂质组学"	5	0
"甲状腺癌" AND "代谢组学"	109	46
"甲状腺癌" AND "血脂"	148	139

The Chinese-language searches strengthened historical patient-tissue support for altered fatty-acid transport and binding in PTC, particularly SLC27A6/FATP6 and FABP3. They did not change the review's central translational conclusion. Most additional records were systemic biomarker studies, narrative reviews, theses, prognostic-signature analyses or preclinical ferroptosis studies.

Bibliographic databases not completed

Institutional access was explored for Embase, Web of Science Core Collection, Scopus, CNKI and SinoMed on 7 June 2026. The access route depended on a local institutional access client that did not provide a stable, reproducible database session. No complete query, result count or export was obtained from these five databases. They are therefore recorded as access attempts, not as searched databases, and their absence is acknowledged as a limitation of this narrative review.

Database	Final status	Treatment in the review
Embase	Access attempted; search not completed	Not counted as searched
Web of Science Core Collection	Access attempted; search not completed	Not counted as searched
Scopus	Access attempted; search not completed	Not counted as searched
CNKI	Access attempted; search not completed	Not counted as searched
SinoMed	Access attempted; search not completed	Not counted as searched

Citation metadata notes

Reference metadata were checked against available DOI, PubMed, CrossRef or publisher-indexed information where available during manuscript preparation. Online ahead-of-print records were cited using the indexed year, volume, issue, page range or article number available at the time of preparation. Early-online 2026 records and records with online/print-year differences should be rechecked at proof stage for updated issue, page or article-number metadata.

Project files used for audit trail

- `literature-review-project/01_search_strategy.md`
- `literature-review-project/searches/pubmed_expansion_summary_v0_2.json`
- `literature-review-project/data/screening_log.csv`
- `literature-review-project/data/screening_log_v0_2_shortlist.csv`
- `literature-review-project/data/extraction_matrix_v0_2_priority.csv`
- `literature-review-project/synthesis/non_pubmed_database_scan_v0_6.md`
- `literature-review-project/searches/multidatabase_search_2026-06-07.md`
